# ‘I went back to being myself’: acceptability of a culturally adapted task-shifted cognitive-behavioural therapy (CBT) treatment for depression (Ziphamandla) for South African HIV care settings

**DOI:** 10.1080/13548506.2019.1566624

**Published:** 2019-01-17

**Authors:** B. Everitt-Penhale, A. Kagee, J. F. Magidson, J. Joska, S. A. Safren, C. O’Cleirigh, J. Witten, J. S. Lee, L. S. Andersen

**Affiliations:** a Department of Psychology, Stellenbosch University, Western Cape, South Africa; b Department of Psychology, University of Maryland, College Park, MD, USA; c HIV Mental Health Research Unit, Department of Psychiatry and Mental Health, University of Cape Town, Cape Town, South Africa; d Behavioral Medicine, Massachusetts General Hospital/Department of Psychiatry, Harvard University, Boston, MA, USA; e Department of Psychology, University of Miami, Miami, FL, USA

**Keywords:** Cognitive behavioural therapy, depression, HIV, adherence, South Africa

## Abstract

There is a need for a culturally adapted, evidence-based, psychotherapy treatment that is effective, acceptable, and feasible for integration into primary care in South Africa. This qualitative study used exit interviews to examine participants’ experiences of an adapted cognitive-behavioural therapy treatment for adherence and depression, task-shifted and delivered by nurses in two peri-urban HIV clinics near Cape Town. Nine semi-structured exit interviews were conducted with isiXhosa-speaking females and analysed using thematic analysis. Overall, participants responded positively to the treatment, viewing it as acceptable and beneficial and as a catalyst to returning to normalcy. Results indicated that participants viewed the treatment as being effective in ameliorating their depressive symptoms and improving their adherence to ART . Additional benefits described included improvements in subjective wellbeing and social and occupational functioning. Several began or resumed employment, an important behavioural indicator of the treatment’s capacity to facilitate positive change and cost saving. Recommendations to improve the treatment included using video material and educating others about depression. These findings have positive implications regarding the acceptability and cultural applicability of the treatment for use in South Africa.

## Introduction

South Africa has the highest human immunodeficiency virus (HIV) burden in the world. Elevated rates of depression have been found among people living with HIV (PLWH) in South Africa (Freeman, Nkomo, Kafaar, & Kelly, 2008). Depression is distressing in and of itself, but it is also associated with HIV disease progression (Leserman, 2008; Myer et al., 2008). Depression is also a risk factor for poor adherence to antiretroviral treatment (ART) (Ammassari et al., 2004; Gonzalez, Batchelder, Psaros, & Safren, 2011; Peltzer, Friend-Du Preez, Anderson, & Anderson, 2010). Failure to adhere correctly is not only associated with poorer prognosis and quality of life, but also carries an increased risk of viral mutation and treatment resistance (Panel on Antiretroviral Guidelines for Adults and Adolescents, Department of Health and Human Services, 2016). Effective treatments for depression that can be integrated into primary HIV care have yet to be identified.

Cognitive-behavioural therapy (CBT) is a structured form of psychotherapy that teaches patients skills to modify their dysfunctional thoughts, beliefs and behaviours (Beck Institute for Cognitive Behavior Therapy, 2016). It is an approach, which has a substantial evidence base for its efficacy in treating depression (Butler, Chapman, Forman, & Beck, 2006; DeRubeis et al., 2005; Hollon & Ponniah, 2010). In this article we report on data from a pilot study, which aimed to evaluate a nurse-delivered CBT intervention for adherence and depression among ART users with depression in South Africa (Andersen et al., 2018). The results of the analysis of the quantitative measures, reported on elsewhere ( Andersen et al., 2018) , were promising regarding the treatment’s usefulness in reducing depressive symptoms, and its acceptability to patients (as demonstrated by high patient retention).

The treatment, called Ziphamandla (meaning ‘Empower Yourself’), was adapted from the Cognitive-Behavioural Therapy Approach for Adherence and Depression (CBT-AD) (Safren, Gonzalez, & Soroudi, 2008). CBT-AD recognises the potentially reciprocal relationship between depressive and illness symptoms and considers that depressive symptoms may interfere with adherence in a medical regime. It integrates CBT treatment for depression with a module on adherence counselling, following the Life-Steps approach (Safren, Otto, & Worth, 1999; Safren et al., 2001). This approach specifically targets adherence by identifying structural and psychological barriers to adherence and implementing strategies to overcome these barriers. Randomised controlled trials of CBT-AD interventions have shown promising results in the United States (Safren et al., 2016, 2009) and Mexico (Simoni et al., 2013). The cultural adaptation of the Ziphamandla treatment included translation into isiXhosa, psycho-education on the process of therapy, contextualising the content, and removal of the module on cognitive restructuring. Furthermore, two additional context-specific steps were included in the Life Steps adherence module, i.e. accessing social support and how to adhere when using substances. See Andersen et al. (2018) for a more detailed description of the adaptations made to the treatment.

Given that mental health professionals are scarce in the public health system, two nurses were trained to deliver the treatment. This task-shifting reflects the World Health Organization, 2007) recommendations for treatments for PLWH in resource-constrained settings. Moreover, other task-shifted psychological interventions, including CBT treatments for depression and ARV-adherence have shown promising results in resource-limited settings (e.g. Abas et al., 2016, 2018; Petersen, Hanass Hancock, Bhana, & Govender, 2014; Rahman, Malik, Sikander, Roberts, & Creed, 2008).

The purpose of the exit interviews conducted with participants post-treatment was to gain insight into participants’ perceptions of the applicability and acceptability of the treatment, including the specific treatment modules, its subjective impact on depressive symptoms, and on ways the treatment could be improved upon.

## Method

### Study context

The Ziphamandla pilot study in South Africa was conducted from September 2011 to June 2012 at two of the busiest primary HIV care clinics in Khayelitsha, a large peri-urban area near Cape Town. In 2011, the last national census indicated that Khayelitsha had a population of 391 749 residents; 10 120 persons per km^2^; with only 44.6% of residents living in formal dwellings (Statistics South Africa, 2011). Official unemployment was 38% and the estimated median monthly income was R1526 ($113) for employed women and R2116 ($157) for employed men (Statistics South Africa, 2011).

### Participants

Fourteen individuals received the treatment and were interviewed for the present study. Five digital recordings were unusable due to technical problems, therefore the remaining nine were analysed. Participants interviewed were all female and isiXhosa-speaking, aged 29–59 years. Individual monthly income ranged from R950 (about $71) to R2060 ($155), with the average being R1134 ($85). Inclusion criteria for the pilot study included being over 18 years old; being fluent in English or isiXhosa; meeting the criteria for a major depressive disorder (as assessed by the Mini International Neuropsychiatric Interview 6.0); having been diagnosed with HIV at least six months prior to data collection; and being enrolled in the ART programme. Exclusion criteria included having received CBT previously; currently being in psychotherapeutic treatment; having had one’s psychotropic medication dose altered or initiated in the previous three months; being actively psychotic or suicidal; or having an uncontrolled neurological problem.

### Procedure

Interviews for the current study took place three months after the treatment was completed. The semi-structured interview guide was informed by the content of the CBT-AD modules and was designed to obtain a broad understanding of each participant’s perception of the treatment and its components. Interviews were conducted by the research assistant in isiXhosa and lasted approximately 30 minutes. Audio-recordings were translated into English and transcribed for analysis. See Andersen et al. (2018) for a full description of the study procedure.

### Treatment

A South African team carried out the adaptation of CBT-AD and two nurses were trained to deliver the treatment. Ziphamandla was shortened from CBT-AD by removing modules such as cognitive restructuring and assimilating relapse prevention into the last module. The degree of training and supervision required to train non-specialists in administering cognitive restructuring was thought to be too labour-intensive and would therefore decrease the feasibility of rollout in primary care settings. This decision was supported by evidence that behavioural interventions, without cognitive therapy, may be at least as effective in treating depression (Dimidjian, et al., 2006; Hollon & Ponniah, 2010).

The treatment ranged from six to eight sessions per patient. It consisted of five modules, namely:

1. Psychoeducation/Motivational Interviewing, which focuses on increasing patient motivation as well as providing information on depression, psychotherapy, and the CBT model;
2. Life Steps for HIV Medication Adherence, which focuses on assisting patients in improving their adherence to ART by identifying potential barriers to adherence and identifying a plan and a back-up plan for overcoming these barriers;
3. Behavioural Activation, which involves identifying appropriate activities and behaviours that give patients a sense of pleasure and accomplishment and assisting participants in engaging in these;
4. Problem-solving, which consists of teaching specific problem-solving strategies to overcome current challenges identified by the patient;
5. Relaxation and Diaphragmatic Breathing Training/Relapse Prevention, which includes teaching deep breathing and relaxation. An audio CD of a guided progressive muscle relaxation exercise is provided. The session also consists of the therapist engaging the patient in a discussion on recognising symptoms of depression and identifying strategies to prevent relapse.

### Data analysis

Braun and Clarke’s approach to thematic analysis was used to analyse the data (Beck Institute for Cognitive Behavior Therapy, 2016). Initially, the data were analysed using an inductive approach (see Boyatzis, 1998), using a detailed reading to generate representative codes. Following this process, we categorised the data according to the treatment modules to which they appeared to correspond. As such, the analysis was characterised by a type of ‘hybrid approach’, incorporating both deductive and inductive processes (see 33). Whilst primarily one researcher undertook the formal analytic process, to provide validation, two other members of the research team also familiarized themselves with the data and provided substantial input at each step.

## Ethics

The study was approved by the University of Cape Town Human Research Ethics Committee (REC REF 035/2010). Pseudonyms have been used in this paper for the participants and the therapists.

## Results

Our results focus on the aspects most pertinent to the aims of the study: the perceived acceptability of the treatment, its subjective impact on depressive symptoms, the perceived applicability of the treatment modules, and ways in which the treatment could be improved.

### Acceptability

The interviews suggested high levels of acceptability of the treatment by participants, who described the treatment as both useful and beneficial to them. Participants reported that the treatment facilitated positive changes in multiple aspects of their lives, including their behaviour, thinking, mood, relationships, self-appraisal and health. Several participants framed these changes as a return to being the person they were before becoming depressed. For example, Khetiwe, age 35, commented, ‘I went back to being myself’.

Participants appeared to perceive the nurse interventionists as acceptable treatment providers or ‘therapists’ and both nurses were regarded very highly. Participants described them as caring, friendly, honest, trustworthy, compassionate, approachable, and good listeners. For example, Babalwa, age 38, stated: ‘She was an approachable person, [sister]. She’s the kind of person that you just go and simply express yourself to her’.

When asked which aspects of the treatment they did *not* find acceptable or useful, none of the participants identified any component. Alternatively, a few emphasised the importance of each aspect. Anathi, age 59, stated: ‘Everything was good about these sessions. Each and every skill that I have learned has helped me’.

### Impact on depression

Overall, the perceived effects on depression are expressed in terms of improvements in various aspects of participants’ lives, including mood and emotional state, health and physical wellbeing, and cognition.

With regards to mood and emotional state, several participants commented that they were feeling ‘better’ following the intervention. Some individuals mentioned more specific emotional changes, including decreased anger and irritability; improved tolerance of being in the company of others; decreased worry and anxiety; and no longer feeling ‘heartbroken’ (Siposethu, age 29).

With regards to health and physical symptoms, several participants reported positive changes. These included decreased pain, fatigue, and dizziness; increased energy; an improved sense of physical well being; and the return of a normal appetite. For example, Anathi, age 59, describes her experience of how her physical symptoms changed following her participation in the treatment:

I was depressed, I was under a lot of stress. […] I had pain on my side. But now I don’t have any pains; I don’t feel any pains in my body.

With regards to cognitive changes, participants reported decreased negative thinking, rumination and forgetfulness; reappraisals of HIV, ART and depressive symptoms (in self and others); a return to/increased faith in God; increased hope for the future; and no longer thinking about suicide. For example, Cebisa, age 35, stated that in terms of her cognitions: ‘Things changed – I wasn’t there thinking painful things, thinking negatively’.

### Applicability of CBT-AD modules

#### Psychoeducation/motivational interviewing

Several participants stated that being diagnosed with and learning about depression facilitated a reappraisal of their experiences and behaviours as a set of symptoms that, importantly, could be treated. Two stated that they had thought depression could only be treated with medication and were surprised to receive counselling instead. Anathi, age 59, expressed the value of learning about depression and its treatment:

The only thing that I would say is that you need – you guys need to educate people around depression. Because if – if I got the help as soon as I needed it, maybe I wouldn’t have been depressed. Had I received help when my daughter passed away, things might have been different.

#### Life steps for HIV medication adherence

Participants attributed changes in adherence to material contained in the Life Steps module – a brief, problem-solving behavioural intervention to address medication adherence. Participants reported improved strategies for taking medication punctually. For instance, several mentioned learning to take their medication at the start of a specific television programme. Other strategies included leaving backup medication with different family members, recruiting family members to assist with medication reminders, and keeping additional medication available.

Participants also reported increased knowledge of the importance of consistent adherence. For example, Siphosethu, age 29, stated ‘I should not think of stopping them, or occasionally taking a break from them’. Relatedly, participants reported changed perspectives on the meaning of medication taking. For example, Cebisa, age 35, reported that her therapist’s normalisation of ART had changed her attitude towards taking her medication: ‘Everyone is taking some kind of treatment. So now I am able to be …okay, with taking my pills’.

#### Behavioural activation

Behavioural activation was strongly reflected in participants’ accounts of the treatment’s benefits. The uptake of activities reported by participants included sport, leisure, social, spiritual and occupational activities, as well as health-related behaviours. Participants commonly reported having returned to activities they had engaged in before becoming depressed, including playing netball, cooking, and going to church. Most participants stated that a key benefit of the treatment was learning the importance of keeping themselves active and busy as a strategy for combating their depressive symptoms. For example, Olwethu, age 37, stated: ‘[I learned that] I should not think too much – I should keep myself busy’. Siposethu, age 29, stated: ‘I don’t contemplate before getting on my feet, I just stand up and do things’.

Moreover, several participants stated that while previously they had been socially isolating themselves, following the intervention they actively pursued spending more time with others. When asked what the most important thing she learned in therapy was, Cebisa, age 35, stated:

I must not always be locking myself behind closed doors. I should be around other people. […] I was always someone who’s always sleeping or is staying indoors or is keeping herself behind closed doors. Now they see that I’m always sitting in the company of people.

#### Problem-solving

Participants’ accounts indicated that they viewed problem solving as highly useful. They spoke at length about the benefits of the advice they received regarding their challenges and the best courses of action. They commented that therapists’ delivery of advice was non-directive, reflecting CBT’s problem-solving approach. Khetiwe, age 35 stated: ‘What I liked about her – it’s the respect that she gave me… She used to show you all the options. And then you would then choose what you will do’.

The treatment reportedly assisted several participants in finding employment, creating income through establishing small businesses, and saving money to further their studies. Significantly, five of the nine participants interviewed who were unemployed at the start of the intervention began working during its course. Four of these participants attributed finding employment to the treatment.

#### Relaxation and diaphragmatic breathing training

A CD with a progressive relaxation exercise in isiXhosa was provided to help participants with their home relaxation practice. Some reported finding it very helpful. For example, Anathi, age 59, described how listening to the CD helped her to manage her physical symptoms:

I used to have a pain in my neck, especially the side of my heart. So, I would listen to the CD and then I would follow what it says. Then after that … I’d find that my body would be much lighter than before.

While Siphosethu, age 29, reported only using the CD at her sister’s home due to lacking electricity in her own, she and three others mentioned that the CD and relaxation exercises had been integral in managing their depressive symptoms.

#### Ways to improve treatment

Participants’ recommendations for improving the treatment included: expanding the programme to include activities (e.g. sewing, handwork) organised by programme staff in-between therapy sessions (Khetiwe, age 35); including visuals (e.g. a video) to complement the audio relaxation CD (Babalwa, age 38); and expanding the programme to educate more people about depression and its treatment (Anathi, age 59). Anathi volunteered to assist with the latter point, commenting:

You could maybe have someone who’s also experienced depression – someone who’s also taken part in the research – someone like me. You can have that person going to the clinic and then they can educate others, speaking from experience – that ‘I’ve also experienced this. But through the therapy that I received everything is fine with me.’

The interviewer also asked for participants’ opinions on the sessions’ frequency and duration. Responses varied, with several commenting that optimal treatment duration depends on individual patients’ needs.

## Discussion

Overall, participants responded positively to the treatment and viewed it as acceptable and beneficial and as a catalyst to return to their normal selves. The results indicated that participants viewed the treatment as having been effective in ameliorating their depressive symptoms and improving their ART adherence. Participants described it as useful and beneficial in multiple ways, including facilitating important improvements in their subjective wellbeing, as well as their social and occupational functioning. Several began or resumed working, an important behavioural indicator of the treatment’s capacity to facilitate positive change and cost saving.

Participants reported positive experiences of each of the Ziphamandla treatment modules and described them all as having been useful. Behavioural activation appears to have been experienced as especially instrumental, reflecting the large body of evidence for the efficacy of behavioural aspects of treatment for depression (Butler et al., 2006; Dimidjian, et al., 2006). Participants also described benefitting a great deal from problem-solving sessions. Several experienced finding employment or informal means of gaining income as a key advantage of the treatment. This finding likely also relates to the socioeconomic demographics of the participants – many were unemployed – and could indicate that problem-solving components have particular value in low-income settings. This mirrors findings from ARV adherence and depression interventions in Zimbabwe (Abas et al., 2016, 2018).

Unlike the original treatment (CBT-AD, Safren et al., 2008), the adapted treatment did not include a cognitive restructuring module. However, it appeared to impact depressogenic thinking indirectly. Participants described experiencing decreases in negative thinking and rumination, as well as more accurate and functional reappraisals of HIV, ART use and depressive symptoms. This finding is promising as it suggests that the treatment can cause changes in thinking without targeting those thoughts directly.

Most of the participants in the study reported that the treatment had helped them with their adherence. The Wisepill data from the parent study indicated improvement, although the small sample size precluded statistical significance (Andersen et al., 2018).

Although not a direct focus of the treatment, another key benefit appears to have been patients improving their social functioning and support outside of therapy. Participants who described their experience of CBT-AD in Berg et al. (2008) U.S. study similarly emphasised the treatment’s impact on helping them to build and increase their use of social support systems.

Limitations included the small sample size, that all participants were women, and that not all the intervention participants’ recordings were able to be analysed for the current study. However, due to the very specific nature of the research question, the range of possible responses to the open-ended interview questions was fairly narrow. For this reason, despite the small sample size, we feel we were able to reach data saturation. We do, however, acknowledge the possibility that other themes could have emerged.

The person who conducted the interviews was not involved in providing the treatment. However, it is possible that participants may have felt uncomfortable expressing criticisms of the treatment, knowing that the interviewer was involved in the study. Another possible contributing factor to why participants did not share any criticisms is that they were treatment naïve (i.e. they had not received previous psychological treatment), therefore they could not compare their experience to other psychological treatments.

Participants were interviewed three months following treatment, thus we did not investigate the treatment’s impact over a longer period. Most of the evidence for CBT’s efficacy in reducing the likelihood of relapse for depression investigates this over several months or a year (for example, see Hollon et al., 2005). There is less evidence for whether these gains are maintained over longer periods, and whether booster sessions or further treatment may be necessary (Westen, Novotny, & Thompson-Brenner, 2004). Future directions include qualitative data collection with the interventionists and assessing multi-level implementation outcomes.

## Conclusion

This study provides support for the Ziphamandla (adapted CBT-AD) treatment’s acceptability and perceived usefulness in targeting adherence and depression in PLWH in South Africa. Participants’ accounts suggest the treatment was able to facilitate behavioural and cognitive changes, which resulted in perceived improvements in their mood, adherence, and overall functioning. In addition, the participants appear to have found the nurses to be acceptable administrators of the intervention, describing their relationships with their therapists in positive terms and characterised by mutual respect. These findings have positive implications for the acceptability of this type of task-shifted treatment in such resource-constrained settings. Next steps include determining the efficacy of the treatment and assessing the feasibility of integrating the intervention into primary HIV care in South Africa.

## References

[CIT0001] Abas M., Bowers T., Manda E., Cooper S., Machando D., Verhey R., … Chibanda D. (2016). ‘Opening up the mind’: Problem-solving therapy delivered by female lay health workers to improve access to evidence-based care for depression and other common mental disorders through the friendship bench project in Zimbabwe. *International Journal of Mental Health Systems*, 10(1), 39–46. 27175215 10.1186/s13033-016-0071-9 PMC4865009

[CIT0002] Abas M., Nyamayaro P., Bere T., Saruchera E., Mothobi N., Simms V., … O’Cleirigh C. (2018). Feasibility and acceptability of a task-shifted intervention to enhance adherence to HIV medication and improve depression in people living HIV in Zimbabwe, a low income country in sub-Saharan Africa. *AIDS & Behavior*, 22(1), 86–101. 28063075 10.1007/s10461-016-1659-4 PMC5758696

[CIT0003] Ammassari A., Antinori A., Aloisi M., Trotta M. M., Monforte A., Monforte A., … Starace F. (2004). Depressive symptoms, neurocognitive impairment, and adherence to highly active antiretroviral therapy among HIV-infected persons. *Psychosomatics*, 45(5), 394–402. 15345784 10.1176/appi.psy.45.5.394

[CIT0004] Andersen L., Magidson J., O’Cleirigh C., Remmert J., Kagee A., Leaver M., … Joska J. (2018). A pilot study of a nurse-delivered cognitive behavioral therapy intervention (Ziphamandla) for adherence and depression in HIV in South Africa. *Journal of Health Psychology*, 23(6), 776–787. 27121977 10.1177/1359105316643375 PMC5081274

[CIT0005] Beck Institute for Cognitive Behavior Therapy (2016). What is cognitive behavior therapy? Retrieved 615, 2017, from https://www.beckinstitute.org/get-informed/what-is-cognitivetherapy/

[CIT0006] Berg C, Raminani S, Greer J, Harwood M, & Safren S. (2008). Participants’ perspectives on cognitive-behavioral therapy for adherence and depression in hiv. *Psychotherapy Research*, 18(3), 271–280. 18815979 10.1080/10503300701561537 PMC3639290

[CIT0007] Boyatzis R. (1998). *Transforming qualitative information: Thematic analysis and code development*. Thousand Oaks, CA: Sage.

[CIT0008] Butler A., Chapman J., Forman E., & Beck A. (2006). The empirical status of cognitive-behavioral therapy: A review of meta-analyse. *Clinical Psychology Review*, 26(1), 17–31. 16199119 10.1016/j.cpr.2005.07.003

[CIT0009] DeRubeis R., Hollon S., Amsterdam J., Shelton R., Young P., Saloman R., … Gallop R. (2005). Cognitive therapy vs medication in the treatment of moderate to severe depression. *Archives of General Psychiatry*, 62(4), 409–416. 15809408 10.1001/archpsyc.62.4.409

[CIT0010] Dimidjian S., Hollon S., Dobson K., Schmaling K., Kohlenberg R., Addis M., … Jacobson N. S. (2006). Randomized trial of behavioral activation, cognitive therapy, and antidepressant medication in the acute treatment of adults with major depression. *Journal of Consulting and Clinical Psychology*, 74(4), 658–670. 10.1037/0022-006X.74.4.658 16881773

[CIT0011] Freeman M., Nkomo N., Kafaar Z., & Kelly K. (2008). Mental disorder in people living with HIV/AIDS in South Africa. *South African Journal of Psychology*, 38(3), 489–500. 10.1080/09540120701426482 18071963

[CIT0012] Gonzalez J., Batchelder A., Psaros C., & Safren S. (2011). Depression and HIV/AIDS treatment nonadherence: A review and meta-analysis. *Journal of Aquired Immune Deficiency Syndrome*, 58(2), 4–13. 10.1097/QAI.0b013e31822d490a PMC3858003 21857529

[CIT0013] Hollon S., DeRubeis R., Shelton R., Amsterdam J., Salomon R., O’Reardon J., … Gallop R. (2005). Prevention of relapse following cognitive therapy vs medications in moderate to severe depression. *Archives of General Psychiatry*, 62(4), 417–422. 15809409 10.1001/archpsyc.62.4.417

[CIT0014] Hollon S., & Ponniah K. (2010). A review of empirically supported psychological therapies for mood disorders in adults. *Depression and Anxiety*, 27(10), 891–932. 20830696 10.1002/da.20741 PMC2948609

[CIT0015] Leserman J. (2008). Role of depression, stress, and trauma in HIV disease progression. *Psychosomatic Medicine*, 70(5), 539–545. 18519880 10.1097/PSY.0b013e3181777a5f

[CIT0016] Myer L., Smit J., Le Roux L., Parker S., Stein D., & Seedat S. (2008). Common mental disorders among HIV-infected individuals in South Africa: Prevalence, predictors, and validation of brief psychiatric rating scales. *AIDS Patient Care STDS*, 22(2), 147–158. 18260806 10.1089/apc.2007.0102

[CIT0017] Panel on Antiretroviral Guidelines for Adults and Adolescents, Department of Health and Human Services (2016). Guidelines for the use of antiretroviral agents in HIV-1-infected adults and adolescents. Retrieved from http://www.aidsinfo.nih.gov/ContentFiles/AdultandAdolescentGL.pdf

[CIT0018] Peltzer K., Friend-Du Preez N., Anderson J., & Anderson J. (2010). Antiretroviral treatment among HIV patients in KwaZulu-Natal, South Africa. *BMC Public Health*, 10(1), 111–121. 20205721 10.1186/1471-2458-10-111 PMC2837855

[CIT0019] Petersen I., Hanass Hancock J., Bhana A., & Govender K. (2014). A group-based counselling intervention for depression comorbid with HIV/AIDS using a task shifting approach in South Africa: A randomized controlled pilot study. *Journal of Affective Disorders*, 158, 78–84. 24655769 10.1016/j.jad.2014.02.013

[CIT0020] Rahman A., Malik A., Sikander S., Roberts C., & Creed F. (2008). Cognitive behaviour therapy-based intervention by community health workers for mothers with depression and their infants in rural Pakistan: A cluster-randomised controlled trial. *Lancet*, 372, 902–909. 18790313 10.1016/S0140-6736(08)61400-2 PMC2603063

[CIT0021] Safren S., Bedoya C. A., O’Cleirigh C., Biello K., Mm P., Mayer K., … Mayer K. H. (2016). Cognitive behavioural therapy for adherence and depression in patients with HIV: A three-arm randomised controlled trial. *The Lancet HIV*, 3(11), e529–e538. 27658881 10.1016/S2352-3018(16)30053-4 PMC5321546

[CIT0022] Safren S., Gonzalez J., & Soroudi N. (2008). *Coping with chronic illness: A cognitive-behavioral therapy approach for adherence and depression*. New York, NY: Oxford University Press.

[CIT0023] Safren S., O’Cleirigh C., Tan J., Raminani S., Reilly L., Otto M., & Mayer K. (2009). A randomized controlled trial of cognitive behavioral therapy for adherence and depression (CBT-AD) in HIV-infected individuals. *Health Psychology*, 28(1), 1–10. 19210012 10.1037/a0012715 PMC2643364

[CIT0024] Safren S. A., Otto M. W., & Worth J. (1999). Life-steps: Applying cognitive-behavioral therapy to patient adherence to HIV medication treatment. *Cognitive and Behavioral Practice*, 6(4), 332–334.

[CIT0025] Safren S. A., Otto M. W., Worth J., Salomon E., Johnson W., Mayer K., & Boswell S. (2001). Two strategies to increase adherence to HIV antiretroviral medication: Life-steps and medication monitoring. *Behavioural Research and Therapy*, 39(10), 1151–1162. 10.1016/s0005-7967(00)00091-7 11579986

[CIT0026] Simoni J., Wiebe J., Sauceda J., Huh D., Sanchez G., Longoria V., … Safren S. A. (2013). A preliminary RCT of CBT-AD foradherence and depression among HIV-positive Latinos on the U.S.-Mexico border: The Nuevo Día Study. *AIDS and Behavior*, 17(8), 2816–2829. 23812892 10.1007/s10461-013-0538-5 PMC3788062

[CIT0027] Statistics South Africa (2011). Main place Khayelitsha. Retrieved 510, 2017, from Statistics South Africa: http://www.statssa.gov.za/?page_id=4286&id=328

[CIT0028] Westen D., Novotny C., & Thompson-Brenner H. (2004). The empirical status of empirically supported psychotherapies: Assumptions, findings, and reporting in controlled clinical trials. *Psychological Bulletin*, 130(4), 631–663. 15250817 10.1037/0033-2909.130.4.631

[CIT0029] World Health Organization (2007). Task shifting: Rational redistribution of tasks among health workforce teams: Global recommendations and guidelines. Retrieved from http://apps.who.int/iris/bitstream/handle/10665/43821/9789?sequence=1

